# Surface Plasmon Resonance-Based Sensing Utilizing Spatial Phase Modulation in an Imaging Interferometer

**DOI:** 10.3390/s20061616

**Published:** 2020-03-13

**Authors:** Roman Kaňok, Dalibor Ciprian, Petr Hlubina

**Affiliations:** Department of Physics, Technical University Ostrava, 17. listopadu 2172/15, 708 00 Ostrava-Poruba, Czech Republic; roman.kanok@vsb.cz (R.K.); dalibor.ciprian@vsb.cz (D.C.)

**Keywords:** surface plasmon resonance, Kretschmann configuration, spatial phase modulation, imaging interferometer, fringe phase shift, sensitivity, aqueous solutions of ethanol

## Abstract

Spatial phase modulation in an imaging interferometer is utilized in surface plasmon resonance (SPR) based sensing of liquid analytes. In the interferometer, a collimated light beam from a laser diode irradiating at 637.1 nm is passing through a polarizer and is reflected from a plasmonic structure of SF10/Cr/Au attached to a prism in the Kretschmann configuration. The beam passes through a combination of a Wollaston prism, a polarizer and a lens, and forms an interference pattern on a CCD sensor of a color camera. Interference patterns obtained for different liquid analytes are acquired and transferred to the computer for data processing. The sensing concept is based on the detection of a refractive index change, which is transformed via the SPR phenomenon into an interference fringe phase shift. By calculating the phase shift for the plasmonic structure of SF10/Cr/Au of known parameters we demonstrate that this technique can detect different weight concentrations of ethanol diluted in water, or equivalently, different changes in the refractive index. The sensitivity to the refractive index and the detection limit obtained are −278 rad/refractive-index-unit (RIU) and 3.6 $\times \;10^{-6}$ RIU, respectively. The technique is demonstrated in experiments with the same liquid analytes as in the theory. Applying an original approach in retrieving the fringe phase shift, we revealed good agreement between experiment and theory, and the measured sensitivity to the refractive index and the detection limit reached −226 rad/RIU and 4.4 $\times \;10^{-6}$ RIU, respectively. These results suggest that the SPR interferometer with the detection of a fringe phase shift is particularly useful in applications that require measuring refractive index changes with high sensitivity.

## 1. Introduction

The surface plasmon resonance (SPR) based sensing, which utilizes the interaction of light with free electrons at a metal-dielectric interface [1] and thus is very sensitive to a large variety of physical/chemical processes at the interface, is the heart of a mature technology with a number of applications in physics [2,3], chemistry [4], biology [5,6], and other fields [7,8,9]. The SPR phenomenon is accompanied by the collective oscillations of free electrons—Surface plasmons (SPs)—That can be excited at the interface by the attenuated total reflection (ATR).

The most efficient way for fulfilling the resonance and thus generating the SPs provides the Kretschmann configuration [2], in which a prism of high refractive index is coated on its base with a thin metal film or a high refractive index glass slide with a thin metal film is attached to the prism. In both cases, the SPs are excited in the metal film by the ATR mechanism, and the field of SPs decays exponentially on both sides of the boundary. Thus, the SPR is extremely sensitive to changes in the refractive index of the surrounding medium (analyte), and is accompanied by a drop in the reflected intensity [4,5,10,11], or by an abrupt phase change [12,13,14,15,16,17,18,19,20].

Optical phase detection techniques [21,22,23] provide a large number of approaches, including heterodyne interferometry [24,25], interferometry with a Mach–Zehnder [12,26,27,28] or imaging interferometer [13], phase quadrature interferometry [29], a phase-shifted polarimetric scheme [30], schemes with a photo-elastic [14] or electro-optic [15] modulator, and a rotating analyzer method [17]. Recently, techniques of spectral interferometry [18,19,20,31,32] and holographic microscopy [33] have emerged as effective tools too. Optical phase detection techniques based on spectral interferometry enable to retrieve abrupt phase changes at several wavelengths simultaneously [18,20], simply by processing a single spectral interferogram. However, the result is very dependent on a technique by which the phase is retrieved. Consequently, single-wavelength interferometric methods are preferable, such as those employing an imaging interferometer with a Wollaston prism and a polarizer, enabling to generate an interference pattern in the spatial domain and to detect a fringe phase shift.

In this paper, we present an imaging interferometer with spatial phase modulation to measure a fringe phase shift due to the SPR phenomenon in the Kretschmann configuration. In the interferometer, a source part with a laser diode (LD), an endlessly single-mode fiber and a collimator are used to generate a beam whose polarized components reflected from an SPR sensing structure are separated by a Wollaston prism. Passing through a polarizer and a lens, the interference of two components is detected by a CCD sensor of a color camera. The refractive index change related to a variable concentration of ethanol diluted in water is converted in the interferometer into a fringe phase shift with a sensitivity of −278 rad/RIU. In addition, an original approach in retrieving the fringe phase shift is applied in experiment and good agreement between experiment and theory is confirmed. With the signal beam related to a *p*-polarized component and reference beam in a common path, the system has excellent noise immunity and high sensitivity reaching for the refractive index value of −226 rad/RIU.

## 2. Experimental Method

**Figure 1:**
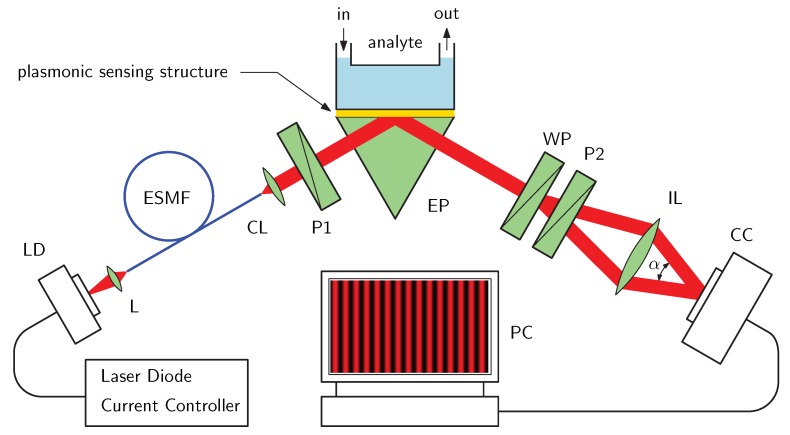
Experimental set-up: an SPR structure in the Kretschmann configuration; laser diode (LD), lens (L), endlessly single-mode fibre (ESMF), collimating lens (CL), polarizer (P), equilateral prism (EP), Wollaston prism (WP), illumination lens (IL), colour camera (CC), personal computer (PC).

Let us consider an experimental set-up, as shows Figure 1, used to measure the phase changes due to the SPR phenomenon for a structure consisting of a high refractive index glass slide with a thin metal film attached to a glass prism by a thin film of index-matching fluid. In the set-up, light from an LD is launched into an optical fiber terminated by an optical element generating a collimated beam. The beam passes through linear polarizer P1 oriented $45^{\circ }$ with respect to the plane of incidence so that both *p*- and *s*-polarized components are generated. These polarized components undergo, owing to reflection from the SPR structure, the amplitude and phase changes that are related to the complex reflection coefficients

(1) $$r_{p,s}=\sqrt{R_{p,s}}\exp\left(i\delta_{p,s}\right),$$

where $R_{p,s}$ and $\delta_{p,s}$ are the reflectances and phase changes, respectively, for both polarizations at the source central wavelength $\lambda_{0}$.

The reflected light passes through a Wollaston prism and the interference of *p*- and *s*-polarized components is attained by using a lens and polarizer P2 oriented $45^{\circ }$ with respect to the plane of incidence. The intensity I(x) as a function of *x* coordinate (a single row of a camera CCD sensor) can be expressed as [13]

(2) $$I\left(x\right)=I_{p}+I_{s}+2\sqrt{I_{p}I_{s}}\cos\left(2\pi \nu_{x}x+\Delta \delta_{ps}\right),$$

where $I_{p}$ and $I_{s}$ are intensity contributions of *p*- and *s*-polarized waves, and $\Delta \delta_{ps}=\delta_{p}-\delta_{s}$ is the phase difference between them. The spatial frequency $\nu_{x}$ is related to the period $T_{x}$ of the generated spatial interference fringes given by

(3) $$T_{x}=\frac{1}{\nu_{x}}=\frac{\lambda_{0}}{2\sin\frac{\alpha }{2}},$$

where α is the angle between the interfering optical beams.

Equation (2) can be rewritten as

(4) $$I\left(x\right)=I_{0}\left[1+V\cos\left(2\pi \nu_{x}x+\Delta \delta_{ps}\right)\right],$$

so that the interference signal S(x) can be expressed as

(5) $$S\left(x\right)=\frac{I(x)}{I_{0}}-1=V\cos\left(2\pi \nu_{x}x+\Delta \delta_{ps}\right),$$

where the visibility *V* of the interference fringes (spatial phase modulation) is given by

(6) $$V=2\frac{\sqrt{I_{p}I_{s}}}{I_{p}+I_{s}}.$$

### Example of Interferograms

**Figure 2:**
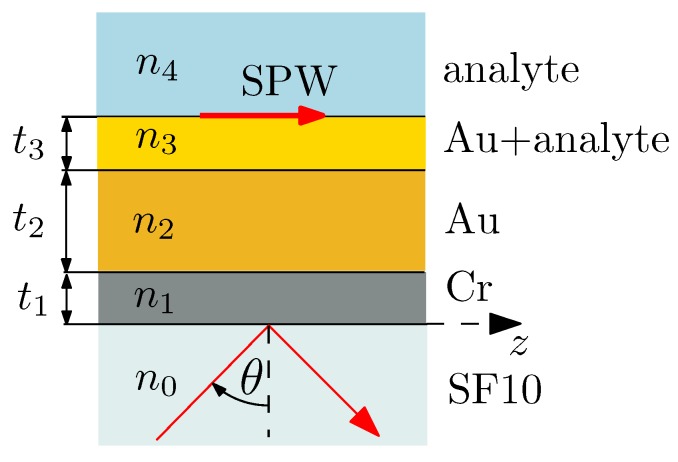
The SPR structure under study.

To model the interference signal S(x) given by Equation (5), we consider an SPR structure (see Figure 2) characterized in detail in a previous paper [34]. It is represented by an SF10 glass slide substrate with an adhesion Cr film of the thickness $t_{1}$ = 2 nm and Au film of the thickness $t_{2}$ = 42.8 nm. The roughness of the gold surface [34] is represented by the effective medium layer (pseudolayer) of the thickness $t_{3}$ = 2 nm with the Au volume fraction *q* = 0.5.

The dispersion properties of all involved materials have been specified [34], and at a wavelength of 637.1 nm of the LD, the refractive indices of the SF10 glass, adhesion Cr film and Au film are $n_{0}$ = 1.7226, $n_{1}$ = 3.6017 + *i*4.3559 and $n_{2}$ = 0.2388 + *i*3.5178, respectively. If the reference analyte is air ($n_{4}$ = 1.00006), the refractive index of the pseudolayer is $n_{3}$ = 0.1639 + *i*2.0548. Similarly, for the first analyte (water, $n_{4}$ = 1.3317), the refractive index of the pseudolayer is $n_{3}$ = 0.1774 + *i*1.9091, and for the second analyte as 30 weight percent (wt%) of ethanol in water ($n_{4}$ = 1.3535), the refractive index of the pseudolayer is $n_{3}$ = 0.1786 + *i*1.8975.

To express the reflectances $R_{p}$ and $R_{s}$, or the intensities $I_{p}$ and $I_{s}$, we evaluate a total transmission matrix M of the SPR structure using [35]

(7) $$M=D_{0}^{-1}\left[\prod_{j=1}^{N}D_{j}P_{j}D_{j}^{-1}\right]D_{N+1}\;,$$

where $D_{j}$ are dynamical matrices, $P_{j}$ are propagation matrices [36], and indices 0 and N+1 refer to the first and last semi-infinite media. In Equation (7), the dynamical matrices are given by

(8) $$D_{j}=\left\{\begin{array}{c} \left(\begin{array}{cc} 1 & 1\\ k_{j} & -k_{j} \end{array}\right)\;\;\mathrm{for}s\mathrm{wave},\\ \left(\begin{array}{cc} 1 & 1\\ \frac{k_{j}}{n_{j}^{2}} & -\frac{k_{j}}{n_{j}^{2}} \end{array}\right)\mathrm{for}p\mathrm{wave}, \end{array}\right.$$

where

(9) $$k_{j}={\left[{\left(n_{j}k_{0}\right)}^{2}-{\left(n_{0}k_{0}\sin\theta \right)}^{2}\right]}^{1/2},$$

with $k_{0}=2\pi /\lambda_{0}$, and the propagation matrices are given by

(10) $$P_{j}=\left(\begin{array}{cc} e^{ik_{j}t_{j}} & 0\\ 0 & e^{-ik_{j}t_{j}} \end{array}\right),$$

where $t_{j}$ is the thickness of *j*-th layer. Using elements $M_{11}$ and $M_{21}$ of the total transmission matrix for *p* and *s* waves, the complex refection coefficients $r_{p,s}$ are expressed as

(11) $$\begin{array}{c} r_{p,s}=\frac{M_{21}}{M_{11}}, \end{array}$$

and the reflectances $R_{p,s}$ are given by

(12) $$R_{p,s}={\left|r_{p,s}\right|}^{2}.$$

**Figure 3:**
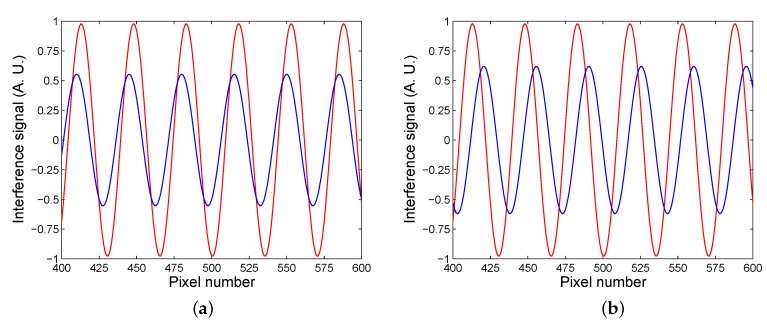
Interference signals for two analytes: water (lower visibility) and air (**a**), 30 wt% of ethanol in water (lower visibility) and air (**b**).

To demonstrate the experimental method, we consider the angle of incidence $\theta =59^{\circ }$ and the period of the spatial interference fringes $T_{x}=175\;\mu$m. If we assume that pixels of the CCD sensor of the camera are separated by 5 μm, Figure 3a shows by the red line the modeled interference signal S(j) as a function of the pixel number *j* for air as analyte. The interference fringes of the visibility approaching one (V=0.979) are obtained, and when the analyte is water, the visibility of the spatial interference fringes is lower (V=0.554) and their phase is changed, as shown in Figure 3a by the blue line, so that the phase shift $\Delta_{\mathrm{SPR}}$, defined as

(13) $$\Delta_{\mathrm{SPR}}=\Delta \delta_{ps}-\Delta \delta_{ps}^{\mathrm{ref}},$$

where $\Delta \delta_{ps}^{\mathrm{ref}}$ is the reference phase shift when the SPR structure is subjected to air, is $\Delta_{\mathrm{SPR}}=0.543$ rad. Similarly, when an analyte is 30 wt% of ethanol diluted in water, the visibility of the spatial interference fringes in comparison with that for air (the fringes are shown by the red line) is lower (V=0.621), as shown in Figure 3b by the blue line, and the phase shift is $\Delta_{\mathrm{SPR}}=-1.353$ rad.

**Figure 4:**
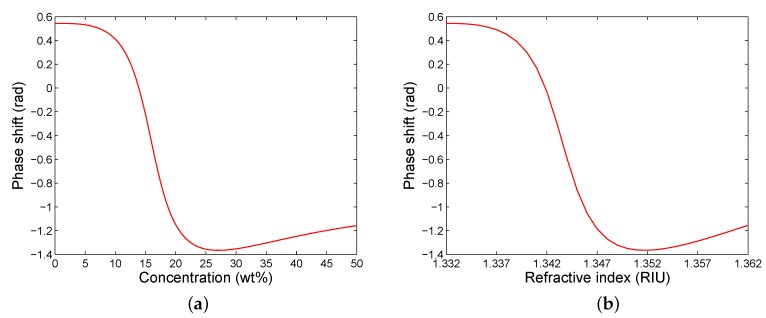
Theoretical phase shift as a function of both concentration of ethanol in water (**a**) and the refractive index of the analyte (**b**).

The theoretical phase shift as a function of the concentration of ethanol diluted in water is shown Figure 4a,b shows the dependence on the refractive index of the analyte. Both curves exhibit an abrupt phase change and the greatest phase shift is for a concentration of 27.06 wt% of ethanol in water and for a refractive index of 1.3517. The non-linear response in Figure 4a indicates variability in the sensitivity to the concentration $S_{c}$, defined as $S_{c}=\delta \Delta_{\mathrm{SPR}}/\delta c$, where $\delta \Delta_{\mathrm{SPR}}$ and δc are the changes in the phase shift and mass concentration of ethanol in water, respectively. The sensitivity $S_{c}$ is with a dip [19] and an extreme value of −0.239 rad/wt%. The phase shift can be approximated in a limited range (approximately from 14.5 to 17.5 wt%) by a linear function so that the sensitivity is constant and reaches $S_{c}=-0.228$ rad/wt%.

Similarly, the sensitivity to the refractive index $S_{n}$, defined as $S_{n}=\delta \Delta_{\mathrm{SPR}}/\delta n$, where $\delta \Delta_{\mathrm{SPR}}$ and δn are the changes in the phase shift and refractive index of the analyte, respectively, is variable. The sensitivity $S_{n}$ is with a dip and an extreme value of –300 rad/RIU. The phase shift can be approximated in a narrow range (approximately from 1.342 to 1.345 RIU) by a linear function, and the sensitivity is constant and reaches $S_{n}=-278$ rad/RIU. If we consider that the SPR phase shift is measured with a precision of 0.001 rad, the detection limit (DL) has a value of 3.6 $\times \;10^{-6}$ RIU.

## 3. Experimental Set-Up

The SPR phase shifts $\Delta_{\mathrm{SPR}}$ for analytes sensed by the structure under test were measured in the experimental set-up shown in Figure 1. The set-up includes an LD (DL-4039-011, Sanyo, Tokyo, Japan) whose output power is adjusted via a protection and strain relief cable (SR9A-ESD, Thorlabs, Newton, MA, USA) by a current controller (LDC201CU, Thorlabs, USA) and it irradiates at a wavelength of 637.1 nm. The light from the LD is launched via optics into an endlessly single-mode optical fiber (FDS-PCF, Fianium, Southampton, UK) and using a collimator (F220FC-B, Thorlabs, USA) and a linear polarizer (LPVIS050, Thorlabs, USA), a collimated beam is incident on an equilateral prism (SF10 glass, Accurion, Goettingen, Germany). An SPR structure represented by an SF10 glass slide substrate with an adhesion Cr layer and Au layer (Accurion, Germany) is attached to the prism using a thin layer of index-matching fluid (Cargille, Cedar Grove, NJ, USA, $n_{D}$ = 1.730).

The beam is incident nearly perpendicularly to the prism face and after the reflection from the SPR structure, it passes through a Wollaston prism (WP10, Thorlabs, USA) and a linear polarizer (LPVIS050, Thorlabs, USA). Two beams from the Wollaston prism are combined by a lens and their interference is attained on a CCD sensor of a color camera (PL-B952U, Pixelink, Ottawa, Canada). USB 2.0 interface of the camera and software PixeLINK Capture OEM enables full computer control of the camera’s features and settings. All the optical parts of the set-up are placed on an optical table (PTQ51508, Thorlabs, USA) to minimize external disturbing effects.

As test analytes, mixed solutions of deionized water and ethanol were prepared. The purity of the ethanol was 99.7% (VWR International, Radnor, PA, USA). The concentrations of ethanol diluted in water were 0 (distilled water), 5, 10, 20, 30, 40, and 50 wt%. The refractive indices of the solutions were independently measured at a wavelength of 589 nm by a digital refractometer (AR200, Reichert, Inc., Depew, NY, USA). The corresponding refractive indices of 1.3334, 1.3359, 1.3387, 1.3426, 1.3504, 1.3557, and 1.3600 were obtained at a temperature of $22{\;}^{\circ }$C.

## 4. Experimental Results and Discussion

**Figure 5:**
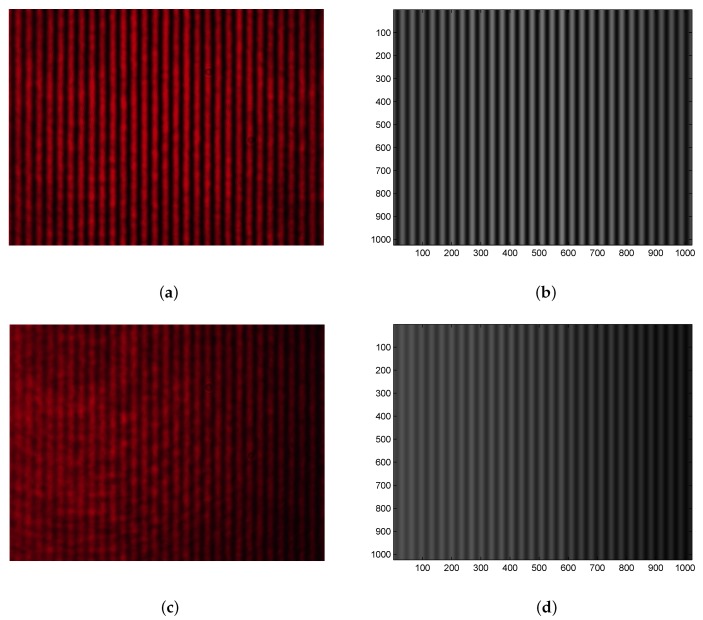
Measurement results: interferograms for air—recorded (**a**) and processed (**b**)—interferograms for water—recorded (**c**) and processed (**d**).

To measure the SPR phase shifts $\Delta_{\mathrm{SPR}}$ for analytes, two interference patterns are acquired in every step by the color camera. The reference interference pattern is related to air as the analyte and the interference image acquired by the camera is shown in Figure 5a. It demonstrates the right orientation of the Wollaston prism and the camera with the fringes perpendicular to the horizontal line. Because the CCD sensor of the color camera has 1024 × 768 pixels, nearly 30 interference fringes acquired are sufficient to retrieve the phase. In the interference pattern, diffraction artifacts due to impurities on optical surfaces are present. They are filtered out, together with the noise, using a simple image processing procedure based on low-pass filtering in the spatial frequency domain. The result of the processing of the red component of the color image is shown Figure 5b and it demonstrates the effectiveness of the filtering.

**Figure 6:**
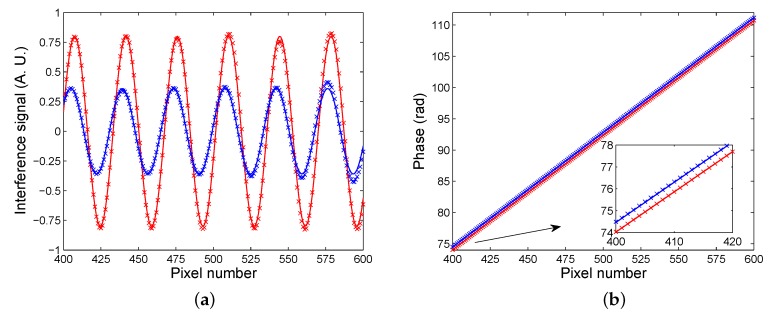
Measured (crosses) and modelled (lines) interference signals for two analytes: water (lower visibility) and air (**a**); retrieved phase as a function of the pixel number for two analytes: water (upper line) and air (**b**).

Figure 6a shows, as crosses, the interference signal obtained from the original signal (pixels at row 384) by removing the unmodulated (reference) signal, which was retrieved using the Fourier-transform low-pass filtering [37]. The reconstructed interference signal is shown in the same figure by the solid curve when the reconstruction of the phase of the signal was done by using a windowed Fourier transform [37]. The interference signal is shown in a narrower range of 400–600 pixels not including the phase errors near the edges. The reconstructed interference signal corresponding to that given by Equation (5) is with the visibility V=0.80. After the injection of the water on the surface of the sensing structure, the interference image shown in Figure 5c was acquired by the color camera. The fringes in the pattern are with lower visibility than in the previous case and they are shifted towards the left side. Figure 5d then shows the result of low-pass filtering in the spatial frequency domain with smooth fringes, and the interference signal is shown in Figure 6a by the crosses. The signal is sampled with a sufficient number of samples, and even if an 8-bit image is stored by the CCD camera, the intensity signal is sufficient to retrieve the phase precisely. The reconstructed phase then enabled to retrieve the interference signal, which is depicted in Figure 6a by the solid curve, and the visibility is V=0.36.

To evaluate the SPR phase shift $\Delta_{\mathrm{SPR}}$ given by Equation (13), we applied an original approach based on a linear fit of the retrieved phase dependence on the pixel number. The corresponding fit for the first analyte (air) is depicted in Figure 6b by a lower line. The fit, characterized by a correlation coefficient as high as 1, is with a slope of 0.1836 rad/(pixel number) and with an intercept of 0.5878 rad. Similarly, the fit corresponding to the second analyte (water), which is shown in Figure 6b by an upper line, is with the same slope and with an intercept of 1.0275 rad. The SPR phase shift for water with respect to the air thus obtained is $\Delta_{\mathrm{SPR}}=0.4397$ rad with a precision of 0.0030 rad.

**Figure 7:**
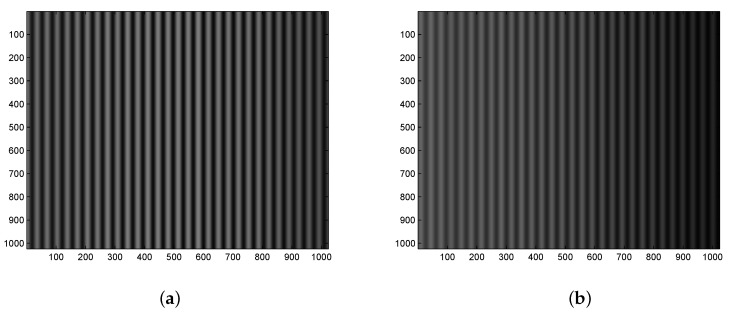
Measurement results: processed interferograms for air (**a**) and 30 wt% of ethanol in water (**b**).

**Figure 8:**
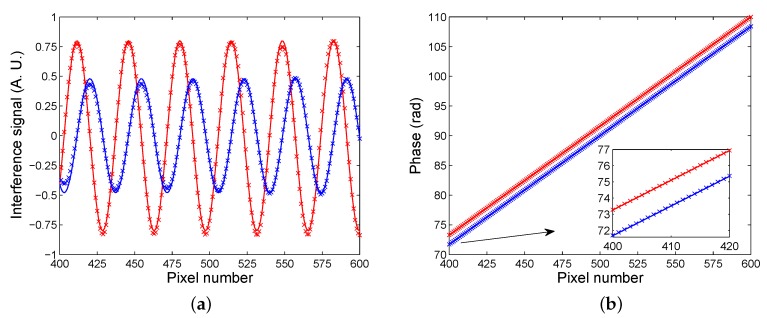
Measured (crosses) and modelled (lines) interference signals for two analytes: 30 wt% of ethanol in water (lower visibility) and air (**a**); Retrieved phase as a function of the pixel number for two analytes: water (lower line) and air (**b**).

Similarly, Figure 7a shows the result of low-pass filtering in the spatial frequency domain with smooth fringes for the reference analyte (air), and Figure 7b shows the same for the measured analyte (30 wt% of ethanol diluted in water). In Figure 8a, the corresponding interference signals with visibilities of 0.80 and 0.48, respectively, are depicted. Contrary to the previous case, the fringes are shifted towards the right side. Applying the above-mentioned approach based on a linear fit of the retrieved phase dependence on the pixel number, the fit for the reference analyte (air), which is shown in Figure 8b by an upper line and is characterized by a correlation coefficient as high as 1, has a slope of 0.1836 rad/(pixel number) and an intercept of −0.1792 rad. In addition, the fit for the measured analyte (30 wt% of ethanol diluted in water), shown in Figure 8b by a lower line, is with the same slope and with an intercept of –1.7652 rad. The corresponding SPR phase shift for 30 wt% of ethanol diluted in water with respect to the air thus obtained is $\Delta_{\mathrm{SPR}}=-1.5860$ rad with a precision of 0.0047 rad.

**Figure 9:**
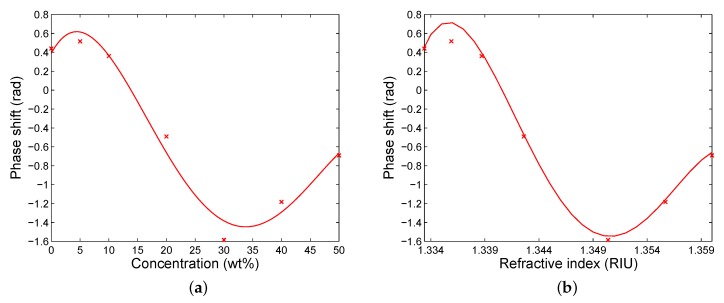
Retrieved phase shift (crosses) as a function of both concentration of ethanol in water (**a**) and the refractive index of the analyte (**b**). Lines are polynomial fits.

The same procedure was applied to the remaining analytes, and the measured phase shift as a function of the concentration of ethanol diluted in water is shown Figure 9a. The dependence is characterized by an abrupt phase change and the greatest phase shift is for a different concentration than that corresponding to the theory (see Figure 4a). This is due to the fact that the real ethanol concentration differs from that at the beginning owing to slow evaporation of ethanol [38] from bottles with the prepared analytes when they are opened. The non-linear response in Figure 9a indicates that the sensitivity to the concentration $S_{c}$ is variable, but it can be considered to be constant in a limited range (approximately from 14 to 21 wt%) and it reaches $S_{c}=-0.107$ rad/wt%.

Similarly, the measured phase shift as a function of the refractive index of the analyte is shown in Figure 9b. The sensitivity to the refractive index $S_{n}$ is with a dip and an extreme value of −236 rad/RIU. This function can be approximated in a narrow range (approximately from 1.340 to 1.345 RIU) by a linear function so that the sensitivity is constant and reaches $S_{n}=-226$ rad/RIU. If we consider that the SPR phase shift is measured with a precision of 0.001 rad, the DL has a value of 4.4 $\times \;10^{-6}$ RIU. Because the refractive index of the analyte is well approximated by a linear dependence on the mass concentration of ethanol in water with a slope of 5.49 $\times \;10^{-4}$ RIU/wt%, the sensitivity $S_{n}$ can also be expressed by means of the sensitivity to the concentration $S_{c}$, and it reaches $S_{n}=-195$ rad/RIU.

## 5. Conclusions

In this paper, a new approach in employing an imaging interferometer with spatial phase modulation to measure a fringe phase shift due to the SPR phenomenon in the Kretschmann configuration has been presented. The interferometer comprises an LD irradiating at 637.1 nm and an endlessly single-mode fiber in an excitation arm, and an SPR sensing structure from which two reflected polarized components of the light beam are separated by a Wollaston prism. Employing a polarizer and a lens, the two components are combined on a CCD sensor of a color camera. In the resultant interference pattern, the refractive index change in a sensed analyte is related to a fringe phase shift. The interference pattern is processed by using low-pass filtering in the spatial frequency domain, and a new approach is adopted to retrieve the SPR phase shift.

The feasibility of the technique has been demonstrated for an SPR structure comprising an SF10 glass prism and a gold-coated SF10 slide with an adhesion film of chromium, when the analytes were aqueous solutions of ethanol. The refractive index changes were measured with a sensitivity of −226 rad/RIU, and the DL reached 4.4 $\times \;10^{-6}$ RIU. These values are limited to a narrow measurement range, approximately from 1.340 to 1.345 RIU, but the refractive index can be measured with similar sensitivity in a different range when an LD irradiating at a different wavelength is employed [20]. Finally, with the signal beam related to a *p*-polarized component and reference beam in a common path, the system has excellent noise immunity and high detection sensitivity, and has the potential to be applied in real-time measurements.
